# The effect of cold atmospheric plasma on diabetes-induced enzyme glycation, oxidative stress, and inflammation; *in vitro* and *in vivo*

**DOI:** 10.1038/s41598-019-56459-y

**Published:** 2019-12-27

**Authors:** Alireza Rezaeinezhad, Pegah Eslami, Hossein Mirmiranpour, Hamid Ghomi

**Affiliations:** 1 Laser and Plasma research Institute, Shahid Beheshti University, G. C., Tehran, Iran; 2 Endocrinology and Metabolism Research Center (EMRC), Valiasr Hospital, School of Medicine, Tehran University of Medical Sciences, G. C., Tehran, Iran

**Keywords:** Applied physics, Plasma physics

## Abstract

Cold atmospheric plasma (CAP) is known as the versatile tool in different biological, and medical applications. In this study, we investigated the effect of cold plasma on diabetes via *in vitro* and *in vivo* assessments. We performed the *in vitro* assay to evaluate the impact of CAP on glycated glutathione peroxidase (GPx) through enzyme activity measurement as a function index and far- and near-UV circular dichroism (CD) and fluorescence analysis as structure indices. The result of *in vitro* assessment showed that the exposure of glycated GPx to plasma causes a considerable increase in enzyme activity up to 30%. Also, the evaluation of far- and near-UV CD and fluorescence analysis indicated a modification in the protein structure. According to obtained result from *in vitro* assessment, *in vivo* assay evaluated the effect of CAP on diabetic mice through analyzing of blood glucose level (BGL), advanced glycation end products (AGEs), antioxidant activity, oxidative stress biomarkers such as malondialdehyde (MDA), advanced oxidation protein products (AOPP), and oxidized low-density lipoprotein (oxLDL), and inflammation factors including tumor necrosis factor (TNF-*α*), interleukin-1 (IL-1), and interleukin-6 (IL-6). The result of *in vivo* experiment also showed a 20% increase in antioxidant activity. Also, the reduction in AGEs, oxidative stress biomarkers, and inflammatory cytokines concentrations was observed. The result of this study revealed that CAP could be useful in diabetes treatment and can be utilized as a complementary method for diabetes therapy.

## Introduction

Plasma is a gas-like system, and considered as the fourth state of the material^1^. It is a partially or wholly ionized gas, which contains neutrals, free electrons, positively and negatively ions, free radicals, active species, molecules and atoms with or without excitation, and UV photons^2^. It can be classified into equilibrium- and non-equilibrium plasma. Also, it may categorize as high-temperature and low-temperature plasma termed as thermal and non-thermal plasma. In the non-thermal plasma, the species are in nonthermal equilibrium. Despite the existence of high-temperature electrons, the overall temperature of plasma, neutrals, ions, and radicals remain constant and close to room temperature; therefore, they are known as cold plasmas, with hindered macro heating of material that they contact with^3^. So, this feature makes the cold plasma suitable for many applications.

Lately, the application of none-thermal atmospheric-pressure plasma or cold atmospheric plasma (CAP) has attracted enormous interest in the medicine, and biology which lead to opening a new approach as the plasma medicine field^4^. Plasma medicine concentrates on increasing the collection of reactive oxygen (ROS) and nitrogen species (RNS), charged particles, UV radiation, and neutral species to cure various pathological conditions. So, reactive oxygen and nitrogen species (RONS) generated by plasmas can play the central roles^5^. Indeed, they can react with cells, tissues, and biological fluids containing proteins, lipids, and carbohydrates^6^. CAP has been widely studied for different applications of plasma medicine, such as various types of cancers, skin diseases, inflammatory disorders, infectious tissues, and production activated liquids^7^.

Diabetes mellitus is a class of chronic metabolic disorders that distinguished by hyperglycemia that is arising from insufficiency of insulin secretion or lack of response to insulin^8^. The control of diabetes, along with induction of oxidative stress, inflammation, and glycation of macromolecules is the leading health challenge and its pervasiveness is increasing in all the world, especially in the developing countries. Currently, more than 400 million people all over the world are suffering from diabetes and this number is expected to reach up to 550 million by 2030^9^. Chronic hyperglycemia by elevating oxidative stress, along with inflammation, may cause to macrovascular and microvascular complications^10^.

Oxidative stress, which has been considered to be a general pathogenic factor of diabetic complications, is related to state which the production of oxidants increased and overcame the endogenous antioxidant capacity, secondary to a loss of the balance between them^11^. Free radical species as oxidants are unstable and highly reactive oxidizing agents because of the existence of single electrons in the outer layer of their orbitals^12^. Free radicals can attack the cellular structure through direct binding with macromolecules such as amino acids, lipids, and nucleic acids which cause oxidative damage to the cell or leading to cell death^13^. Among various types of free radicals, oxygen free radicals are the most common in aerobic organisms, which often known as ROS^14^. The cellular redox balance can be examined by the analysis of both antioxidant and oxidant biomarkers^15^. ROS have a very short lifetime (10^−6^–10^−9^ seconds), so it is not easy to detect their presence^16^. Among the oxidant biomarkers, hydrogen peroxide (H_2_O_2_) as a ROS molecule can be measured in body fluids due to their long half-life^14^. So oxidative stress can be evaluated by measuring the final products of ROS as biomarkers of oxidative stress, including oxidized products of lipid, protein, and low-density lipoprotein (LDL) such as the malondialdehyde (MDA), advanced oxidation protein products (AOPP), and oxidized low-density lipoprotein (oxLDL), respectively^17^.

Hyperglycemia not only leads to excessive free radicals but also reduces antioxidant activity through glycation and conformation change of antioxidant enzymes^18^. Under normal physiological conditions, the cellular redox balance depends on several antioxidant systems^13^. Glutathione peroxidase (GPx), as a protein, is an essential intracellular antioxidant enzyme decomposing H_2_O_2_ into the water, mainly in mitochondria and sometimes in the cytosol^19^. So, glycation of GPx denatures its structure and consequently, decreases its enzymatic activity, reduces H_2_O_2_ decomposition, which leads to an accumulation of hydrogen peroxide, and ultimately increase oxidative stress. In general view, the elevation of blood glucose level (BGL) results in long-term modification of lipids, proteins, and nucleic acids, which causees forming advanced glycation end-products (AGEs)^20^. AGEs are the final products of a sequence of chemical reactions which are triggered due to the bonding of the carbonyl group in the carbohydrate like glucose with the free amino group in a biomolecule such as protein, without enzyme intervention^14,21^. The formation of AGEs, and oxidative stress are intertwined; the increasing AGEs may lead to oxidative stress, and vice versa; ROS may facilitate AGEs formation^20^.

Also Suggested that the induction of oxidative stress and AGEs, due to hyperglycemia, leads to inducing inflammatory cytokines like tumor necrosis factor (TNF-*α*), interleukin-1 (IL-1), and interleukin-6 (IL-6) that can investigate as the biomarkers of hyperglycemia^22^.

Several studies have been conducted on using the cold plasma in diabetes mellitus complications treatment, directly, such as wound healing in case of diabetic rats, and indirectly such as nano-sized clinoptilolite production by the cold plasma, and its effect on reducing hyperglycemia in rats^2,5,23^. Also, in recently promising study has been reported after diabetic wound treatment by the cold plasma, the levels of superoxide dismutase (SOD), glutathione peroxidase (GPx), and catalase (CAT) elevated, and the result of CAP on wound healing revealed the plasma role in upregulating cytokines secretion (as an important part of inflammatory system) such as IL-1, and IL-6^5,24^. In literature, yet there is no exhaustive and cumulative investigation into the effect of CAP on oxidative stress, AGEs, and irregular inflammatory system.

So, in this study, both *in vitro* and *in vivo* assessments helped us to evaluate the effect of CAP, as a noninvasive and safe method, on hyperglycemia, glycated protein, oxidative stress, and inflammatory system. For *in vitro* assay, the glycated GPx exposed to argon plasma jet, and function and structure of the enzyme analyzed. Also, the effect of the cold plasma on diabetic mice was investigated through an *in vivo* assessment with analyzing antioxidant activity, oxidant parameters, inflammation factors, and BGL.

## Results

### In vitro

#### OES

**Figure 1:**
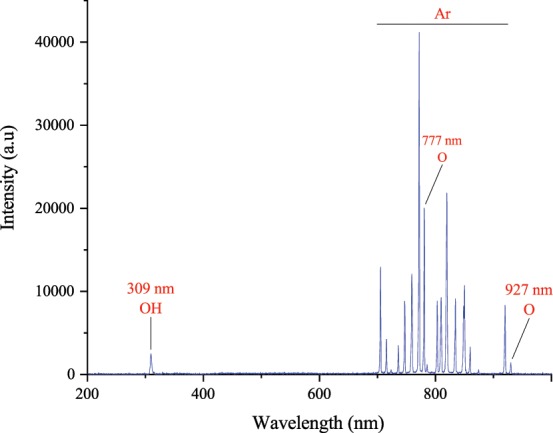
The emission spectrum of Ar plasma in air.

The emission spectrum of argon plasma jets in the air medium has been shown in Fig. 1. The spectrum line of argon plasma jet indicates the existence of hydroxyl radical, nitrogen, argon, and oxygen species.

#### Effect of the cold plasma on enzyme activity

**Figure 2:**
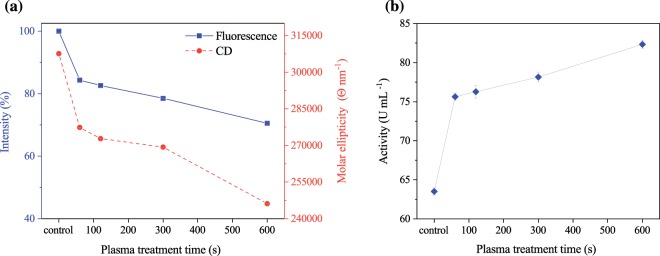
(**a**) Fluorimetry intensity, dichroism spectrum, and (**b**) activity of plasma treated glycated GPx for different plasma treatment times (60–600 seconds) (p < 0.001). Error bars stand for standard deviation.

The activity of the enzyme (as a protein) is affected by protein conformational changes such as glycation. So, for studying the impact of CAP on protein conformation, enzyme activity can be analyzed. Figure 2 shows the effect of the argon plasma jet treatment on GPx activity for different plasma exposure times. As inferred from Fig. 2, enzyme activity is a function of the cold plasma treatment time. Such that, after 600 seconds of treatment, enzyme activity increased from 63.51 (for 100% glycated GPx) to 82.33 U mL^−1^.

#### Fluorimetry analyze

The effect of CAP on glycated GPx structure was investigated by fluorimetry analyze (Fig. 2). According to results, the improvement of protein denaturation, which is a reverse process of enzyme glycation has been occurred through receiving plasma treatment. Figure 2 shows that with the enhancement of plasma exposure time from 60 to 600 seconds, the naturation of GPx increased so that the 600-second imposing argon plasma jet to the solution leads to about 30% naturation.

#### Circular Dichroism (CD) analyze

The ellipticity measurement is the most popular method used for monitoring protein structural changes due to destabilizing agents such as glycation. The results of molar ellipticity analysis show a significant change after the cold plasma treatment for different plasma exposure times (Fig. 2); such that, by increasing treatment time, the molar ellipticity decrease, and after 600 seconds of treatment, its amount reduced from 307617 to 246139 Θ nm^−1^.

### In vivo

#### Effect of CAP on BGL, AGEs, antioxidant activity, and H_2_O_2_ concentration

**Figure 3:**
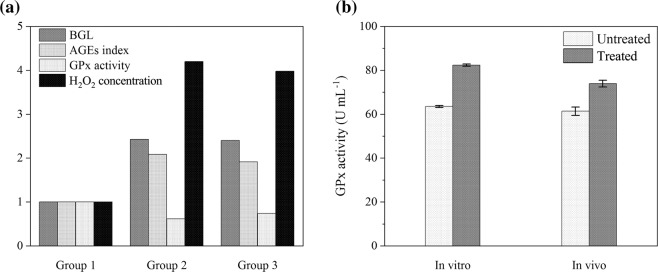
(**a**) BGL, AGEs index, GPx activity, and H_2_O_2_ concentration for each group (group1: normal mice, group 2: diabetic mice, group 3: plasma treated diabetic mice) normalized to that in group 1 (p < 0.001). (**b**) Accordance of *in vitro* and *in vivo* assessments.

An increase in BGL leads to producing AGEs due to direct exposure of lipid or protein to sugar. Moreover rising glucose level results in enzyme glycation, a decrease in antioxidant activity, and oxidants accumulation. Table 1 illustrates the mean values and standard divisions of BGL, AGEs index, GPx activity, and H_2_O_2_ concentration for normal mice (group 1), diabetic mice (group 2), and CAP treated diabetic mice (group 3). Figure 2 also shows the mentioned indices in each group, normalized to that in group 1. As indicated in Table 1 and Fig. 3, BGL of plasma treated diabetic mice is lower than diabetic mice, and its amount is reduced from 391.5 to 388.1 mg dL^−1^ for 600 seconds applying argon plasma. Also, by comparing the AGEs index of Group 2, and 3, it is clear that the plasma has a positive effect on the reduction of this index. Moreover, the obtained data revealed that CAP is effective in antioxidant activity enhancement. Such that the plasma treatment leads to increasing GPx enzyme activity, and decreasing H_2_O_2_ concentration. On average, a 20% increase in enzyme activity was observed at 600 seconds plasma exposure time that this result was in accordance with *in vitro* ones (Fig. 3).

**Table 1:** Mean values and standard deviations of BGL, AGEs index, GPx activity, and H_2_O_2_ concentration (p < 0.001) for normal mice (group 1), diabetic mice (group 2), and CAP treated diabetic mice (group 3).

Variable	Group 1		Group 2		Group 3	
	Mean	SD	Mean	SD	Mean	SD
BGL (mg dL^−1^)	161.40	3.85	391.53	23.19	388.13	19.10
AGEs (mL^−1^)	3.89	0.22	8.11	0.21	7.46	0.16
GPx activity (U mL^−1^)	130.37	2.28	79.97466	1.90	96.38	1.58
H_2_O_2_ (mmol L^−1^)	30.64	2.33	128.67	3.81	121.96	2.48

#### Effect of CAP on oxidative stress

**Figure 4:**
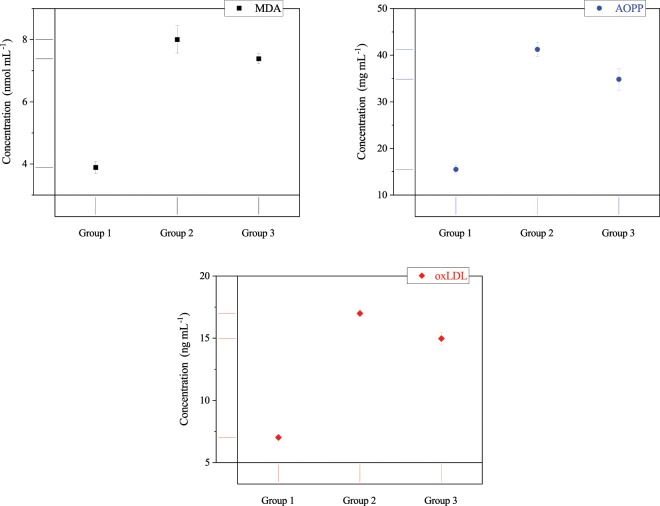
MDA, AOPP, and oxLDL concentrations (p < 0.001) as biomarkers of oxidative stress (group 1: normal mice, group 2: diabetic mice, group 3: plasma treated diabetic mice). Error bars stand for standard deviation.

MDA, AOPP, and oxLDL indices, as the oxidant biomarkers of oxidative stress, are shown in Fig. 4. It indicates that the MDA concentration is reduced by more than 0.6 nmol mL^−1^ after using the cold plasma. Also, it is notable that the cold plasma has decreased AOPP concentration by more than 15%. Furthermore, the impact of CAP treatment on oxLDL can be distinguished as a reduction of 2.0 ng mL^−1^ in group 3. Overall, these results indicate that the cold plasma treatment plays an important role in the reduction of oxidative stress and related oxidant biomarkers.

#### Effect of CAP on inflammation factors

**Figure 5:**
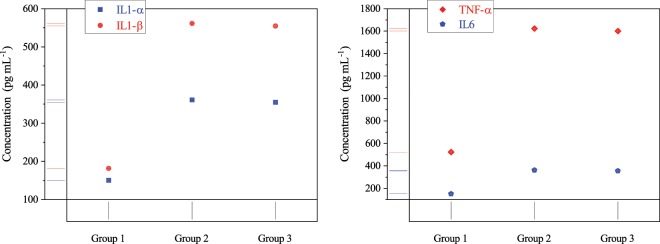
Inflammation factors concentrations (p < 0.001) for normal mice (group 1), diabetic mice (group 2), and plasma treated mice (group 3). Error bars stand for standard deviation.

CAP impact on IL-1 (*α*, and *β*), TNF-*α*, and IL-6 concentrations were studied (Fig. 5). The result shows that the inflammatory factors of plasma treated group are closer to the normal ones (group one) relative to untreated mice and its reduction amounts for all indices was about 1%. These results confirm the cold plasma influence on the reduction of inflammation.

#### The overall effect of the cold plasma treatment on diabetic mice

The ANOVA test is a general analyze for studying and comparing mean vectors of the three groups. So, for the exact determination of pairwise differences for each variable based on the ANOVA test, post-hoc tests can be considered (Tables 2–4). Obtained results from Tukey-HSD test with 95% family-wise confidence level for BGL, AGEs index, GPx activity, and H_2_O_2_ concentration has been shown in Table 2, oxidant biomarkers concentrations (oxidative stress) has been illustrated in Table 3, and inflammation factors indices has been presented in Table 4. According to the confidence interval resulting from Tukey’s post-test, the possibility of comparing variable mean between two groups has been provided that this is a criterion to discuss about significant differences in three groups. For all factors, the means difference between group one and two (1–2: group 1 minus group 2) are negative (except to GPx) due to enhancing variable after STZ injection. So, the diabetes-induced process was carried out successfully. Also, for groups two and three, all mean difference (3–2) is negative except to GPx, as a variable was descended after CAP treatment. Therefore, this method is effective for complications arising from diabetes. By comparing the means difference (1–2), and (1–3) together, the same results can be obtained.

**Table 2:** Obtained result from Tukey-HSD test for BGL, AGEs index, GPx activity, and H_2_O_2_ concentration with 95% family wise confidence level (p < 0.001).

Variable	Groups	Difference	95% family-wise confidence level	
			Lower bound	Upper bound
BGL	3–2	−3.40	−18.91	12.11
	1–2	−230.13	−245.64	−214.62
	1–3	−226.73	−242.25	−211.22
AGEs	3–2	−0.66	−0.83	−0.48
	1–2	−4.22	−4.40	−4.05
	1–3	−3.57	−3.74	−3.39
GPx activity	3–2	16.41	14.69	18.13
	1–2	50.39	48.67	52.11
	1–3	33.98	32.26	35.70
H_2_O_2_	3–2	−6.71	−9.33	−4.09
	1–2	−98.03	−100.65	−95.41
	1–3	−91.32	−93.94	−88.70

**Table 3:** Obtained result from Tukey-HSD test for oxidative stress biomarkers concentrations with 95% family wise confidence level (p < 0.001).

Variable	Groups	Difference	95% family-wise confidence level	
			Lower bound	Upper bound
MDA	3–2	−0.62	−0.88	−0.36
	1–2	−4.12	−4.37	−3.86
	1–3	−3.50	−3.76	−3.24
AOPP	3–2	−6.44	−7.95	−4.92
	1–2	−25.76	−27.27	−24.24
	1–3	−19.32	−20.83	−17.81
oxLDL	3–2	−2.02	−2.34	−1.70
	1–2	−9.97	−10.28	−9.65
	1–3	−7.95	−8.27	−7.63

**Table 4:** Obtained result from Tukey-HSD test for inflammations factors indices with 95% family wise confidence level (p < 0.001).

Variable	Groups	Difference	95% family-wise confidence level	
			Lower bound	Upper bound
IL-1*α*	3–2	−6.23	−8.64	−3.83
	1–2	−210.53	−212.93	−208.12
	1–3	−204.29	−206.70	−201.89
IL-1*β*	3–2	−6.96	−9.63	−4.29
	1–2	−380.01	−382.68	−377.37
	1–3	−373.05	−375.72	−370.38
TNF-*α*	3–2	−21.63	−24.98	−18.27
	1–2	−1100.38	−1103.73	−1097.03
	1–3	−1078.75	−1082.10	−1075.40
IL-6	3–2	−7.12	−9.47	−4.77
	1–2	−210.73	−213.079	−208.39
	1–3	−203.61	−205.96	−201.27

## Discussion

In this study, the effect of CAP on glycated GPx as *in vitro* and diabetic mice as *in vivo* is investigated. To the best of our knowledge, we pioneered to introduce a basic nonclinical research for evaluating CAP impact either on glycated protein or diabetes.

**Figure 6:**
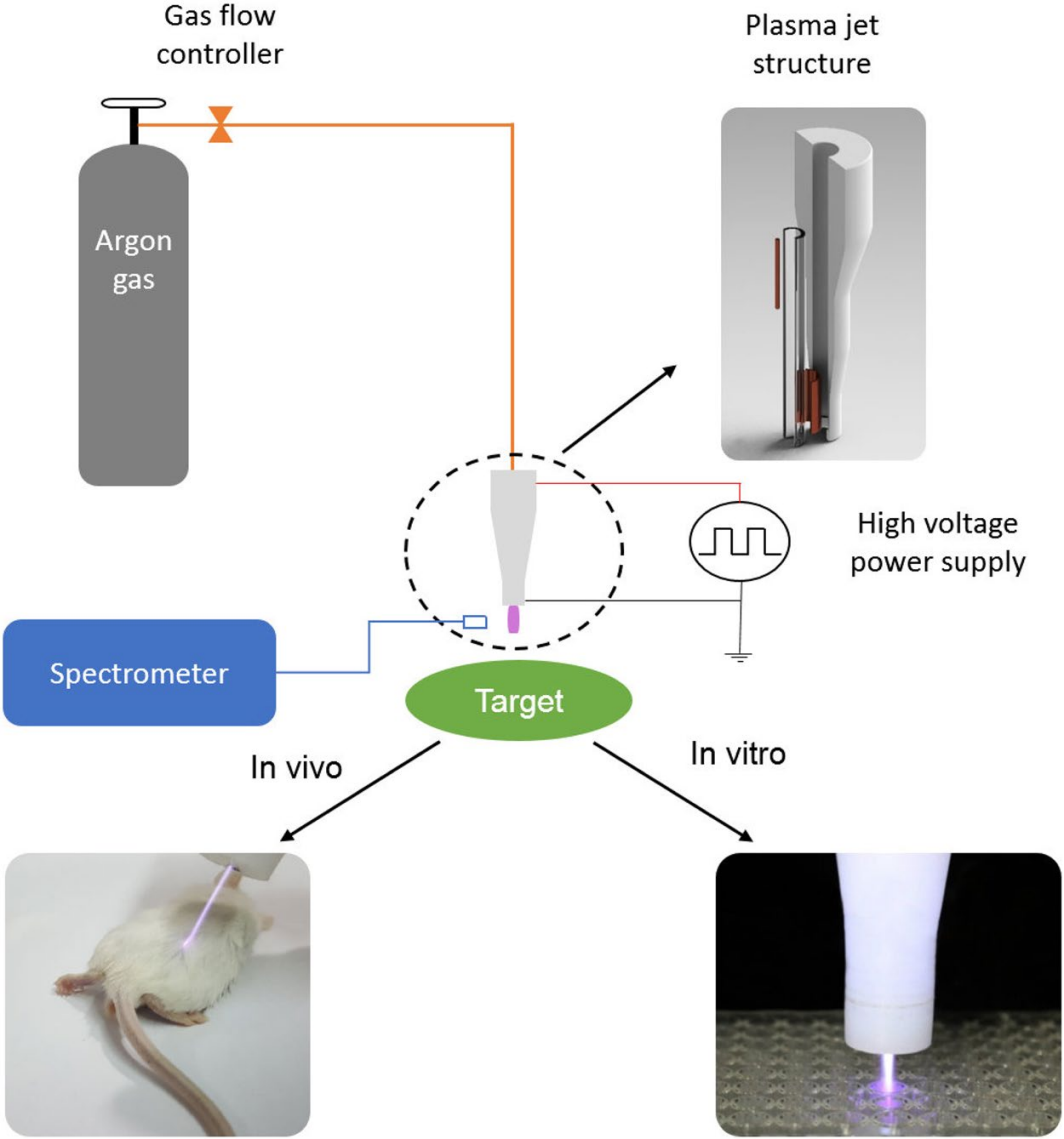
The schematic of *in vitro* and *in vivo* assessments.

As illustrated in Fig. 6, the argon plasma jet is containing both hydroxyl, and singlet oxygen radicals. RONS has two-edged sword nature in biology, as it could be either beneficial or harmful to biological systems depending on plasma properties (such as plasma source type, mode of energy dissipation into the system, feeding gas, plasma plum, and types and concentrations of reactive species), type of organism, and exposure time^3,6^. However, in the present study, the physiological characteristic of plasma is used as a positive induction to modify denatured protein structure and enzymatic activity. The results of the *in vitro* assessment showed that plasma treatment led to a modification of protein conformation and enzymatic activity improvement (Fig. 2). It is clear that these factors strongly correlated; such that the improvement of protein conformation directly affects the enzyme activity. Also, these effects have been augmented over CAP treatment time.

Many studies have been focused on the interaction of plasma with various proteins. Attri *et al*. have shown that CAP could lead to a modification in protein oxidation, dimerization, nitration, and dehydrogenation of amino acids^25^. On the other hand, Takaei *et al*. reported that nitration, hydroxylation, sulfonation, amidation, and disulfide linkage formation in amino acids could occur during the cold plasma treatment^26^. For example, Choi *et al*. concluded that the reduction in the fluorescence intensity of the plasma-treated lysozyme was due to the modification of the Trp group or Trp surroundings^27^. Altogether, quenching of intrinsic fluorescence and CD spectra intensity of denatured GPx after exposure to CAP could be the result of modifications of amino acids and variations in the secondary and tertiary structures in the vicinity of the modified amino acids. Such that these modifications shifted the denatured GPx toward its native structure and subsequently enzymatic activity enhanced.

Also, *in vivo* result is consistent with data obtained from *in vitro* assay, which this accordance is notable (Fig. 3). The slight difference observed in enzymatic activity between *in vitro* and *in vivo* assessments could be due to the direct treatment of glycated GPx in exposure to plasma plum and short-lived reactive species. In the gas phase, plasma generates different types of ions and radicals that can interact with an aqueous solution to produce biodiverse reactive species with short/long lifetime in a liquid phase. Some of the short-lived RONS such as hydroxyl (OH·), superoxide (${{\rm{O}}}_{2}^{-}$), and singlet oxygen (O·) have been measured in an aqueous solution^28^. These species decay to produce long-lived species such as ozone (O_3_), hydrogen peroxide (H_2_O_2_), nitrite (${{\rm{NO}}}_{2}^{-}$), and nitrate ions (${{\rm{NO}}}_{3}^{-}$)^28^. So, in direct treatment, the glycated GPx is affected by either short-lived species or long one, whereas glycated GPx in the bloodstream could receive only the effect of the long-lived species.

Reduction of H_2_O_2_ concentration in the blood of affected mice shows an increase in GPx activity, which implies a modification in protein structure, due to the fact that activity and structure of a protein are correlated to each other. As a result of this phenomenon, the oxidative stress reduced and inclined the body to the balanced state between oxidants and antioxidants. The decrease in the oxidative stress led to the reduction in the oxidation of proteins, lipids, and lipoproteins. Therefore, the oxidative stress biomarkers, including MDA, AOPP, and oxLDL diminished after plasma treatment.

Also, It is widely proved that low-density lipoprotein (LDL) particles are extremely susceptible to oxidative damage^29^. In other words, ROS could attack to LDLs and oxidize the lipids and protein components of LDL particles^30^. oxLDL plays a vital role in the initiation, development, and progression of atherosclerosis, and causes endothelial inflammation, and endothelial dysfunction^31,32^. Uptake of oxLDL by lectin-like oxLDL receptor-1 (LOX1), the receptor on endothelial cells, and macrophages via the scavenger receptors leads to the promotion of foam cell formation and increases the release of inflammatory cytokines including IL-1, IL-6, and TNF-*α*^30,31,33,34^. So, in our work, the decrease in oxLDL due to the reducing of either oxidant or oxidative stress can be related to the declined expression of inflammatory cytokines such as IL-1, IL-6, and TNF-*α*.

It is well known that the presence of RONS, as the essential cellular second messengers, are necessary for the physiological performance of insulin but, it turns out to that RONS is also associated with resistant of body cells to insulin^35^. That is (to say), they can have a physiological or pathological effect on insulin functions^6^. It seems the extension of RONS along with induction of oxidative stress alters the structure of the protein that acts as insulin signaling molecules or otherwise affects insulin signaling pathways in a harmful way^35^. Therefore, the decrease in oxidative stress could reduce the resistance of cell and body to insulin and improve insulin performance. One of the results in the following of these phenomena could be the reduction in the BGL.

On the other hand, rising BGL content leads to non-enzymatic glycation process or Millard reaction involves unstable Schiff’s bases formation through the attachment of carbonyl group of reducing sugar (such as glucose) to free amino group in proteins. As a result, rearrangement to stable ketoamine products, or Amadori compounds occur^36^. These products can undergo further reactions, such as cyclization, and condensation, to form AGEs^37^. It is well known that reduction of BGL diminishes non-enzymatic glycation process of protein or lipid as a result of exposure to glucose. These phenomena result in decreasing of AGEs concentration of the plasma-treated diabetic mice.

Moreover, the effect of the cold plasma light also should be considered. The mitochondria rotary motor that is known as ATP synthase is the most efficient molecular motor^38^. It consists of two parts: a largely hydrophilic and a hydrophobic part^39^. So, a local change in nanoscopic interfacial water viscosity alters the performance and the dynamics of the nanomotor system^38^.

Mitochondria produces both ROS and ATP compounds that ROS can affect the intramitochondrial space. These species can improve hydrophilicity and thus the viscosity of the interfacial water layers bound to exposed surfaces of intramitochondrial. So oxidative stress situation leads to an increase in interfacial viscosity, along with an increase in viscous friction, which contributes to the drop in ATP production related to performance reduction of the rotary motor^38^.

It has been shown that the nanoscopic interfacial water layers structure can be modified by different wavelengths of laser lights such as 633 and 670 nm^38,40,41^. The modification involves expansion of the volume and reduction of viscosity, particularly on hydrophilic surfaces, that are known as promoting high viscosities^42,43^. As illustrated in Fig. 1, CAP spectrum includes different emissions; in ultraviolet, infrared, and visible regions. These emissions might be effective in modulation of interfacial water, promotion of mitochondrial ATP synthase and procession of glucose metabolism. So, the reduction in BGL can be the result of emitted light-spectra by CAP. This reduction might contribute directly to reduced GPx glycation (enhancement of antioxidant activity) and AGEs index, and might have an indirect outcome in the favorable change of MDA, AOPP, oxLDL, and inflammation factors indices.

Metformin is primarily utilized for Type 2 diabetes treatment as well as oral anti-diabetes agent^44^. Although, it has considerable benefits, including low risk of hyperglycemia, weight neutrality, cost-effective, and possible cardiovascular advantages, up to 25% of patients treated with metformin suffer metformin-associated gastrointestinal (GI) side-effects, and 5% are unable to tolerate metformin due to the severity of these side effects^45^.

As a conclusion, the result of this study revealed that the CAP could be effective in the modification of glycated GPx (as denatured protein) and improvement of its enzymatic activity. Also, the obtained data showed that the cold plasma treatment has a positive effect on the reduction of oxidative stress, AGEs, and inflammatory cytokines concentrations in diabetic mice. So, the cold plasma treatment, as a novel and complementary method, can be utilized with metformin for diabetes therapy to lower metformin dosage and reduce metformin’s side effects.

## Materials and Methods

### Materials

#### Atmospheric Pressure Plasma Jet (APPJ) device and instrumentation

As illustrated schematically in Fig. 6, the APPJ consisted of a dielectric, powered, and ground electrodes, and a high voltage power supply. A Pyrex tube (L: 150 mm, ID: 4 mm, OD: 6 mm) utilized, as the dielectric barrier and the nozzle. A copper rod (L: 30 mm, D: 1 mm) and a thin copper cylindrical tube (L: 15 mm) used as the powered and ground electrodes, respectively. The powered electrode was inserted in the tube from one end, while the other tube end was surrounded by the ground electrode, such that the distance between the nozzle tip and the lower edge of the ground electrode was 5 mm. The plasma was generated by a 10 kHz pulsed DC power supply with an amplitude up to 10.0 kV. Also, argon gas with a purity of 99.999%, and 3 l min^−1^ flow rate used as feeding gas.

#### Reagents and filter

GPx (9013-66-5), phosphate buffered saline (PBS) (P5368), and glucose (G7021) were obtained from Sigma-Aldrich Co (USA). 0.22 µm filter was supplied from Millipore Corporation, Billerica, MA (USA).

#### Assay kits

Activity assay kit of GPx (D-89075) was purchased from Biocore Diagnosik Ulm GmbH Co (Germany). Quantity assay kits of IL-1*β* (MBS175967), AGEs (MBS704846), AOPP (MBS263319), MDA (MBS264973), and oxLDL (MBS2512757) were acquired from MyBioSource Co (USA). Quantity assay kits of IL-1*α* (BMS611), IL-6 (LMC0061), and TNF-*α* (BMC607-3) were obtained from Termo Fisher Chem Co (USA). Also, quantity assay kits of H_2_O_2_ (E-BC-K102) and glucose (81692) were supplied from Elabscience and Crystal Chem companies (USA), respectively. Streptozotocin (STZ) (S0130) and nicotinamide (NA) (N0636) were purchased from Sigma-Aldrich Co (USA).

### Methods

#### In vitro

##### Optical Emission Spectroscopy (OES)

CAP applications mostly are based on the generation capability of sufficient amounts of various reactive species. So, optical emission spectroscopy (OES) was used to analyze the existence, and intensity of any species. For this purpose, an ocean optic HR 2000 spectrometer was employed. The spectral range was chosen from 200 to 11000 nm with an optical resolution of 0.5 nm. Plasma optical emissions recorded at 20 mm distance from plasma stream. According to the atomic spectra database lines, the recorded spectrum was analyzed and different species were determined.

##### Preparation of glycated GPx solution

A pure solution of GPx (10 mg mL^−1^) was prepared by combining the protein with phosphate buffered saline (PBS) at pH 7.4, and a 50 mM l^−1^ glucose solution was made by mixing pure glucose with PBS. An aliquot of the prepared GPx solution was combined with glucose solution and named glycated GPx. Then, pure GPx and glycated GPx samples were filtered under the sterilized condition and incubated for 16 weeks at 37 °C^46,47^. At the end of 16th week, Glycated GPx was treated by cold plasma, and an aliquot of each of the three solutions (pure GPx, glycated GPx, and treated glycated GPx) was taken, and kept at −80 °C until could be analyzed by three methods: Circular Dichroism (CD), fluorometry, and activity assay^48^.

##### CAP treatment

CAP treatment was performed by direct exposing the prepared Glycated GPx to the plasma jet stream (Fig. 6). Two mL of the glycated enzyme solutions loaded in a 2 mL 96-well plate, and the the cold plasma applied at the 15 mm distance from the the solution surface with four different durations (60, 120, 300, and 600 seconds). Also, the untreated enzyme solution considered as the control sample.

##### Function analyze

Measurement of GPx Function was performed by related activity assay kit, enzymatic colorimetric method, and Tecan’s Sunrise absorbance microplate reader (Switzerland). The enzyme activity was measured as U mL^−1^.

##### Fluorescence spectroscopy

Each sample with a concentration of 0.5 mg mL^−1^ was analyzed by Shimadzu spectrofluorometer RF-5000 (Japan, Kyoto). Wavelengths of 350, and 440 nm were considered as excitation, and emission wavelengths, respectively. The results have been presented as percentage^46,49^.

##### CD analyze

A CD Spectropolarimeter (JASCO-810, Jasco, Tokyo, Japan) was applied to study the structure of the samples with a concentration of 0.1 mg mL^−1^. The spectra were modulated and obtained, as units of mean residue molar ellipticity [Θ] (mdeg cm^2^ dmol^−1^), based on the average weight of the amino acids (112.4). The equation [Θ] *λ* = (Θ× 112.4)× cl^−1^ indicated the molar ellipticity (calculations were performed at 25 °C)^50,51^.

#### In vivo

##### Subjects

Sixty male BALB/c type mice aged 6 weeks with an average body weight of 30 g (bought from Pasteur Institute of Iran) were housed in a vivarium under controlled condition (a temperature of 23 ± 3 °C, and a relative humidity of 50 ± 10%) with a 12:12 h light-dark cycle, and had free access to rat chow and water ad libitum. After one week of acclimatization to these conditions, forty-five mice which had showed favorable growth were selected and randomly allotted into three groups (n = 15): (1) Nondiabetic control (Normal ones without any interference), (2) Diabetic control (Diabetic ones involved by STZ), (3) Affected diabetics (Diabetic ones involved by STZ, and affected by cold plasma). Diabetes was induced in mice (groups 2, and 3) by intraperitoneal (i.p.) injection of a single dose of STZ (50 mg kg^−1^) that after 15 min followed up by administration of nicotinamide (NA) (120 mg kg^−1^). One week after injection, serum glucose ≥200  g  L^−1^ (checked by glucose assay kit (Crystal Chem Co, USA)) was considered as diabetes^52^. The animal ethics review committee (the Biological Research Institute of Cognitive, and Brain Sciences, Shahid Beheshti University) approved the study protocol under the international guidelines for the care and use of laboratory animals^53^.

##### CAP treatment

*In vivo* assay involved treatment of 15 diabetic mice (group 3) by APPJ. According to the information obtained from the analysis of *in vitro* processes, each mouse treated for 600 seconds. The back skin of the mice was chosen for treatment (Fig. 6).

##### Sampling

One week after exposure to plasma, blood samples of mice were obtained from the vein of their orbits. Related serum samples of each group were prepared to detect the biochemical parameters. Recent preparation was accomplished during 15 min centrifugation of blood at 5000 × g, clot separation, and storage at 70 °C for further studies.

##### Oxidants analyze

Oxidant parameters, including AGEs, AOPP, MDA, and oxLDL were measured by enzyme-linked immunosorbent assay (ELISA) techniques according to their assay kits and ELISA reader apparatus (MR-96A, Mindary Co) (Germany). H_2_O_2_ was detected by the colorimetric method, quantity assay kit, and Tecan’s Sunrise absorbance microplate reader (Switzerland).

##### Antioxidant analyze

The function of GPx was determined according to the method described in *in vitro* section.

##### Inflammatory factors analyze

Inflammatory parameters, including IL-1*α,* IL-1*β*, IL-6, and TNF-*α*, were assayed by ELISA method, detection kits, and ELISA reader apparatus (MR-96A, Mindary Co, Germany).

##### Glucose analyze

The enzymatic colorimetric method, related kit, and Tecan’s Sunrise absorbance microplate reader (Switzerland) were used to determine serum glucose of mice.

### Data processing

#### Statistical analyze

All data are expressed as the mean value of 45 mice blood analyze results. In this study, 11 variables were investigated for each of the groups. For this purpose, statistical analysis were performed through the ANOVA test with 0.001 significant level (p < 0.001) by the dplry package of R software (R-3.5.2 version). Also, based on the ANOVA test, the posthoc test (Tukey-HSD test) was applied for pairwise comparison of the three groups means.
